# COVID-19 in Bangladesh: Data deficiency to delayed decision

**DOI:** 10.7189/jogh.10.010342

**Published:** 2020-06

**Authors:** Samin Huq, Raaj Kishore Biswas

**Affiliations:** 1 Child Health Research Foundation, Dhaka, Bangladesh; 2 Transport and Road Safety (TARS) Research Centre, School of Aviation, University of New South Wales, Australia

Lower middle-income countries are not heavily presented in global media on COVID-19. Bangladesh, a densely populated nation, have conducted only 1759 tests in last one month with 9 reported deaths. The decisions of cluster-wise lockdown or social distancing, or even preparing the health system to respond to the pandemic are made without the availability of adequate data. This is fuelling panic in local community and giving an obscure picture in the global data.

With major discussions around COVID-19 pandemic focused on China, USA and Europe, few media reports have acknowledged that it has also silently paved its way into lower middle-income countries. In Bangladesh, as per the 5^th^ of April 2020, only 88 diagnosed cases and 9 deaths have been associated with COVID-19 with first case detected on the 8th of March [1]. As of the 1st of April, the number of tests conducted was 1759 since the detection of the first COVID-19 case.

In a country of 164 million and with 155 898 overseas arrivals between the 8^th^ of March and the 5^th^ of April, which included some hard-hit countries such as Italy, a total of less than 3000 tests in the first 4 weeks of transmission were likely insufficient to illustrate the viral transmission in Bangladesh [1]. Although scarcity of test kits and lack of awareness from the general public certainly contributed to the crisis, Bangladesh is one of the few countries which had more than two months to prepare for COVID-19 crisis. It could have been both aware and prepared, because of its close business ties with China.

Due to the shortage of test kits, authorities had to set strict eligibility parameters for the test. Thus, only those with direct contact with foreign returnees were tested. There was a refusal to consider the possibility of any community transmission, which created confusion. Furthermore, there was a rather strong opposition to the development of rapid diagnostic kits on diagnostics attributes such as sensitivity and specificity, without careful consideration on affordability and subsequent accessibility. This problem has contributed to a reduced availability of baseline information on the degree of transmission of COVID-19 in Bangladesh. The situation forced the government to enforce measures without being able to rely on substantial data.

In Bangladesh, the COVID-19 crisis was therefore a game of “catching up”. The government has undertaken measures iteratively, in response to the situational development in the country. It promoted hand hygiene and other protective measures, such as social distancing, airport screening, quarantine wherever necessary, and restricted movement during government holidays [2]. As the final step, the government decided on a nation-wide shutdown, with army deployed to ensure a proper lockdown.

The crisis has already started in the form of limited availability of disinfectants and personal protective equipment in the health care facilities. This led to elevated perceived risk on COVID-19 infection in the hospitals. Many facilities are now refusing to admit patients who suffer from other routine complications. Lack of testing also limited quarantines of close contacts of the suspected cases. These resulted to country-wide fear and an avalanche of reports on deaths with pneumonia/flu-like symptoms.

The fear soon turned to a panic, as some villages or entire local areas were locked down by local authorities over untested possible COVID -19 related deaths. The only testing facility during the first 3 weeks of the crisis (the 8^th^ to the 25^th^ March) received over 420 629 phone calls to request testing, but was only able to test 0.3% of those who raised a concern over their status [3]. This shows the extent of the burden that the authorities in Bangladesh had to deal with. The lack of information fuelled the panic.

On the 24^th^ of March, the government extended testing to multiple facilities following a supply of 40 500 test kits from China. However, the rush towards testing in absence of a careful planning within these facilities for sample collection and diagnosis created more confusion. This is because some facilities with test kits did not have laboratories with biosafety level 2 or no trained personnel who could adhere to a standardized protocol [4].

The crisis worsened with increases in prices due to shortages of essential items. Such a scenario can result in limited affordability in a capitalistic economy, where families are dependent upon the single-wage earners. In the era of free-flowing online information, “discoveries” based on weak evidence and related comments had a massive influence on the decision making at the individual level. This led to, for example, overstocking of medications such as hydroxychloroquine and azithromycin, which created an artificial scarcity.

Partial lockdown aimed at social distancing may allow virus containment for a period of time [5]. However, it comes at a cost as resource-intensive and demands meticulous contact testing. Furthermore, widespread testing becomes difficult to replicate in a resource-limited health care infrastructure. Bangladesh has inequitable distribution of health care workforce concentrated in the major urban areas. This is coupled with one of the lowest availability of health care workforce in the world, with only 7.4 skilled workers per 10 000 population [6]. Aligning the situation with the dimension of access by Penchasky and Thomas [7], limited availability coupled with reduced accessibility in the peri-urban and rural health care settings may contribute to a health care catastrophe in addressing the control of COVID-19.

**Figure:**
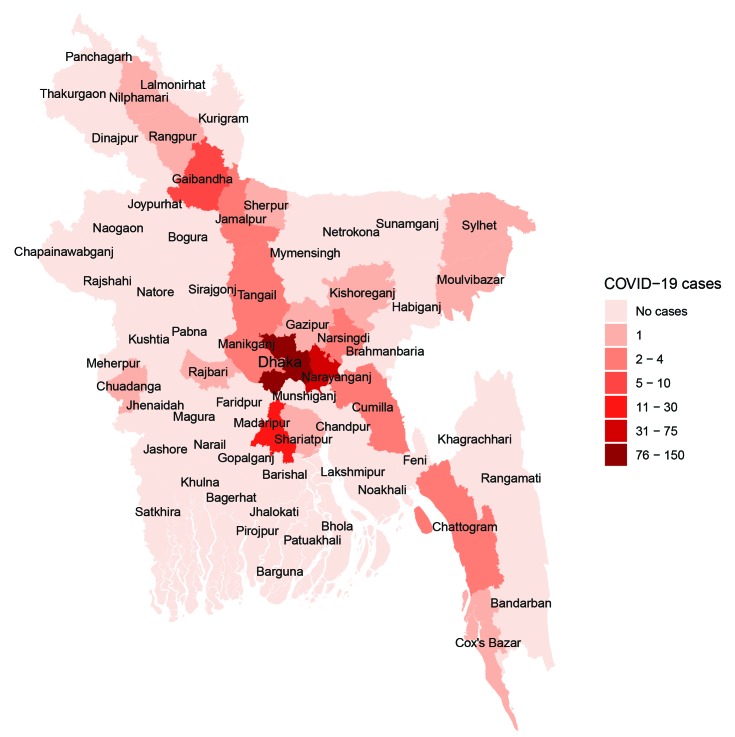
Photo: The distribution of positive COVID-19 cases across the districts of Bangladesh (8 April 2020), data source: https://iedcr.gov.bd/.

The recent surge of daily testing has managed to uncover more cases and traces of transmission at the community level. Despite the surge, the testing rate is too low to uncover the true burden of COVID-19 in Bangladesh, considering the population size (13 tests per million) [8]. On the other hand, the surge on daily testing has only managed to expose some hotspots which were already known to be the densely populated areas of the capital. Therefore, the recent opening of the manufacturing industries, with subsequent closure due to criticism, contributed to migration to the overcrowded areas of the city [9]. Such initiatives can lead to a dual burden of risk transmission, to both the rural community and the urban community. This happens at a greater pace, owing to the mass migration of the factory workers.

Also, decision making based on the current prevalence of COVID-19 can undermine the issue of the health system preparedness without a careful understanding of the dynamics of COVID-19 evidenced worldwide. It can include premature lifting of restricted movement and sanctions and lower concentration of testing and contact tracing in the rural areas. These can impact not only the health system planning and response in terms of financial input, but also appropriate service delivery and required supply to address the COVID-19 due to the changing dynamics of population density in the event of reduced sanctions. Additionally, it can lead to a surge on heightened risk of exposure due to minimized health response priorities, efforts and initiatives - such as contact tracing and testing in the rural areas and considering their movement to the urban areas.

While pandemics hardly give opportunities to many countries to prepare, developed nations will have the data to at least trace their progress and assess their position on the curve. Unfortunately, the curve for Bangladesh seems flat with only one case detected in a period of 72 hours (the 28^th^ to the 30^th^ March). This could be because either Bangladesh is extremely lucky, or this scarcity of databased on appropriate testing now obscures an ominous death toll that is to follow.

## References

[1] Directorate of Health Services Goverment of Bangladesh. Situation analysis of novel COVID-19 2020. Available: https://corona.gov.bd/press-release. Accessed: 31 March 2020.

[2] Institute of Epidemiology, Disease Control and Research. General information COVID-19 2020. Available: https://www.iedcr.gov.bd/index.php/component/content/article/73-ncov-2019. Accessed: 1 April 2020.

[3] Directorate of Health Services Goverment of Bangladesh. Daily and Monthly Call Report. 2020.

[4] Bangladesh DoHSGo. Situation analysis of novel COVID-19. 2020. Available: http://16263.dghs.gov.bd/report/report.php. Accessed: 25 March 2020.

[5] Hellewell J Abbott S Gimma A Bosse NI Jarvis CI Russell TW Feasibility of controlling COVID-19 outbreaks by isolation of cases and contacts. Lancet Glob Health. 2020;8:e488-96. 10.1016/S2214-109X(20)30074-7 32119825 PMC7097845

[6] World Health Organization. The Global Health Observatory data repository. 2020. Available: https://apps.who.int/gho/data/node.main.HWFGRP_0020. Accessed: 31 March 2020.

[7] Penchansky R Thomas JW The concept of access: Definition and relationship to consumer satisfaction. Med Care. 1981;19:127-40. 10.1097/00005650-198102000-00001 7206846

[8] Worldometer. Coronavirus Cases. 2020. Available: https://www.worldometers.info/coronavirus/coronavirus-cases/. Accessed: 5 April 2020.

[9] Tribune D. RMG workers asked to go back from factory gates. 2020. Available: https://www.dhakatribune.com/bangladesh/2020/04/05/rmg-workers-asked-to-go-back-from-factories-gates. Accessed: 5 April 2020.

